# Molecular Basis for the Evolution of Species-Specific Hemoglobin Capture by Staphylococcus aureus

**DOI:** 10.1128/mBio.01524-18

**Published:** 2018-11-20

**Authors:** Jacob E. Choby, Hanna B. Buechi, Allison J. Farrand, Eric P. Skaar, Matthew F. Barber

**Affiliations:** a Department of Pathology, Microbiology, and Immunology, Vanderbilt University Medical Center, Nashville, Tennessee, USA; b Vanderbilt Institute for Infection, Immunology, and Inflammation, Vanderbilt University Medical Center, Nashville, Tennessee, USA; c Graduate Program in Microbiology and Immunology, Vanderbilt University, Nashville, Tennessee, USA; d Institute of Ecology and Evolution, University of Oregon, Eugene, Oregon, USA; Nanyang Technological University

**Keywords:** *Staphylococcus aureus*, evolution, heme transport, hemoglobin, host-pathogen interactions, iron acquisition

## Abstract

Metals are a limiting resource for pathogenic bacteria and must be scavenged from host proteins. Hemoglobin provides the most abundant source of iron in the human body and is required by several pathogens to cause invasive disease. However, the consequences of hemoglobin evolution for bacterial nutrient acquisition remain unclear. Here we show that the α- and β-globin genes exhibit strikingly parallel signatures of adaptive evolution across simian primates. Rapidly evolving sites in hemoglobin correspond to binding interfaces of IsdB, a bacterial hemoglobin receptor harbored by pathogenic Staphylococcus aureus. Using an evolution-guided experimental approach, we demonstrate that the divergence between primates and staphylococcal isolates governs hemoglobin recognition and bacterial growth. The reintroduction of putative adaptive mutations in α- or β-globin proteins was sufficient to impair S. aureus binding, providing a mechanism for the evolution of disease resistance. These findings suggest that bacterial hemoprotein capture has driven repeated evolutionary conflicts with hemoglobin during primate descent.

## Abstract (precis)

During infection, bacteria must steal metals, including iron, from the host tissue. Therefore, pathogenic bacteria have evolved metal acquisition systems to overcome the elaborate processes mammals use to withhold metal from pathogens. Staphylococcus aureus uses IsdB, a hemoglobin receptor, to thieve iron-containing heme from hemoglobin within human blood. We find evidence that primate hemoglobin has undergone rapid evolution at protein surfaces contacted by IsdB. Additionally, variation in the hemoglobin sequences among primates, or variation in IsdB of related staphylococci, reduces bacterial hemoglobin capture. Together, these data suggest that S. aureus has evolved to recognize human hemoglobin in the face of rapid evolution at the IsdB binding interface, consistent with repeated evolutionary conflicts in the battle for iron during host-pathogen interactions.

## INTRODUCTION

Animals possess a variety of molecular factors that effectively sequester essential metals from invasive microbes, contributing to an innate immune function termed nutritional immunity (1, 2). Iron, as a critical cofactor for many host and bacterial enzymes, has provided the paradigm for our current understanding of nutritional immunity. Since the discovery of iron limitation by egg white ovotransferrin in the 1940s (3), the mechanisms underlying nutritional immunity and bacterial iron scavenging have been the subject of intense study (4). Many vertebrate-associated bacteria harbor high-affinity uptake systems targeting heme, an abundant iron-containing porphyrin cofactor (5).

The most abundant source of heme iron in the mammalian host is hemoglobin, which mediates oxygen transport within circulating erythrocytes. The predominant adult hemoglobin consists of a tetramer containing two α-globin and two β-globin protein subunits, each of which binds a single heme molecule for the coordination of oxygen. The Gram-positive bacterium Staphylococcus aureus is well adapted to the human host and is a leading cause of skin and soft tissue infections, endocarditis, osteomyelitis, and bacteremia (6). To acquire iron during infection, S. aureus has evolved a high-affinity hemoglobin binding and heme extraction system, termed the iron regulated surface determinant (Isd) system (7). Following the lysis of proximal erythrocytes via secreted bacterial toxins, the released hemoglobin is captured by receptors at the S. aureus cell surface (8, 9). The Isd system of S. aureus in part consists of cell wall-anchored IsdB and IsdH, which bind hemoglobin and haptoglobin-hemoglobin, respectively (9, 10).

We and others have shown that IsdB is the primary hemoglobin receptor for S. aureus and critical for pathogenesis in murine infection models (9, 11–13). Additionally, IsdB is highly expressed in human blood (14) and a promising vaccine target (15), underscoring its importance in human disease. IsdB extracts heme from hemoglobin, and heme is subsequently passed across the cell wall and into the cytoplasm for degradation by the heme oxygenases IsdG and IsdI, liberating iron (16–20). Underscoring the importance of IsdB for pathogenesis, heme is the preferred iron source of S. aureus during murine infection (21). The cell wall-anchored IsdABCH proteins share between one and three NEAT (near transporter) domains for the coordination of hemoglobin or heme. IsdB NEAT1 binds hemoglobin while NEAT2 binds heme, tethered by an intervening linker (22). Consistent with the adaptation of S. aureus to colonize and infect humans, we previously found that S. aureus IsdB binds human hemoglobin more effectively than mouse hemoglobin, the common laboratory animal used to model S. aureus infection (13). These results suggest that hemoglobin variation among mammals dictates effective heme acquisition by S. aureus and other Gram-positive bacteria.

Previous work has demonstrated that pathogens can promote a rapid adaptation of host immunity genes through repeated bouts of positive selection (23–25). While adaptation during such evolutionary conflicts can take many forms, theoretical and empirical studies indicate that an elevated rate of nonsynonymous to synonymous substitutions in protein-coding genes is often indicative of recurrent positive selection (26, 27). To date, most empirical studies of host-pathogen “arms races” have focused on viruses (28–31). Recently, we showed that the transferrin family of iron-binding proteins has undergone extremely rapid evolution in primates at protein surfaces bound by iron acquisition receptors from Gram-negative bacteria (32, 33). These findings are consistent with the existence of a long-standing evolutionary conflict for nutrient iron, whereby mutations in iron-binding proteins that prevent bacterial scavenging protect the host from infection and are favored by natural selection. While these studies have expanded our understanding of how pathogens shape the evolution of host genomes, they also raise the question of whether other components of nutritional immunity might be subject to similar evolutionary dynamics.

In addition to its role as the principal bloodstream oxygen transporter, hemoglobin has provided an important biological model for diverse areas of the life sciences. Elegant studies have illustrated how hemoglobin variation underlies multiple instances of adaptation to high altitudes in diverse vertebrate taxa (34–37). Hemoglobin alleles have also likely been subject to the balance of selection in human populations, where mutations that produce sickle-cell disease also confer resistance to severe malaria (38) and have reached high frequencies in regions where malaria is endemic. Despite its long history of study, the consequences of hemoglobin evolution for vertebrate nutritional immunity remain unclear. In the present study, we set out to investigate the evolution of hemoglobin family proteins in primates and determine whether primate hemoglobin evolution impacts the ability to sequester heme iron from bacterial pathogens.

## RESULTS

### Parallel signatures of positive selection in primate hemoglobins at the IsdB binding interface.

**FIG 1:**
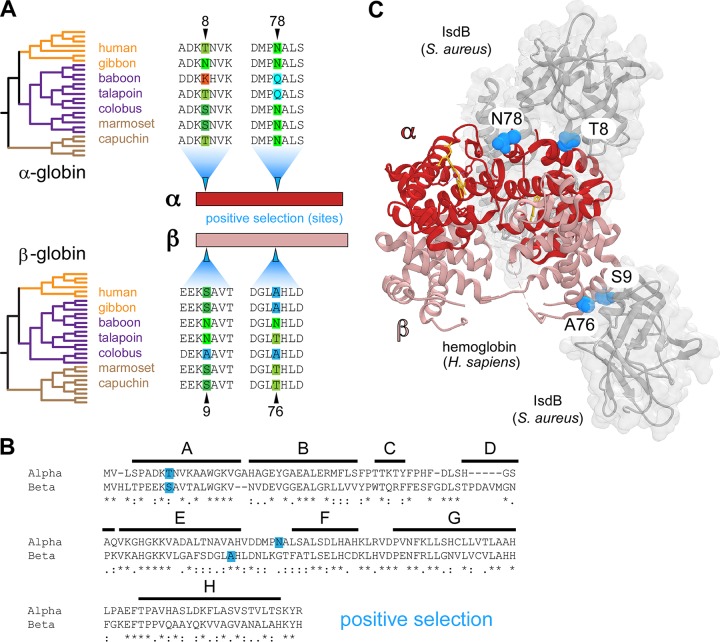
Parallel signatures of positive selection among primate hemoglobins at the bacterial IsdB binding interface. (A) Species phylogenies for the 27 α-globin and 30 β-globin orthologs analyzed (left). Alignments from representative species across hominoid (orange), Old World monkey (purple), and New World monkeys (brown) are shown, the full phylogenetic trees are displayed in Fig. 3 and 4. Amino acid sites under positive selection were identified in α-globin and β-globin (blue arrows) using PAML with both species and gene trees (posterior probabilities, >0.95). (B) Amino acid alignment of human α-globin and β-globin proteins. The position of conserved helices A to H are shown, and identity between globins is noted as conserved (*), highly similar (:), and similar (.). (C) The residues of α-globin (red) and β-globin (salmon) under positive selection (blue spheres) at the interface of hemoglobin capture by Staphylococcus aureus IsdB (gray) (PDB 5VMM).

To investigate how natural selection has shaped hemoglobin diversity in simian primates, orthologs of the α- and β-globin genes were cloned and sequenced from primate cell lines as well as compiled from publicly available databases. In total, 27 α-globin and 30 β-globin orthologs were assembled for phylogenetic analyses using the PAML and HyPhy software packages (Fig. 1A; see also Materials and Methods), which use nonsynonymous and synonymous substitution rates to infer signatures of positive selection. Because globin genes have been shown to undergo gene conversion, which can distort inferred phylogenetic relationships (39, 40), all analyses were performed using both a well-supported species tree as well as gene trees generated using PhyML. All tests detected significant evidence of positive selection acting on both α- and β-globins using both species and gene phylogenies (see Data Set S1 in the supplemental material). Multiple analyses repeatedly identified two sites in α- and β-globins exhibiting strong signatures of positive selection (Fig. 1A). It became apparent that these rapidly evolving sites localized to similar regions of the α- and β-globin proteins, specifically, the N-terminal A helix and the hinge region between the E and F helices (Fig. 1B). In fact, the two sites exhibiting signatures of selection in the α- and β-globin A helices are at homologous positions. These parallel signatures of selection between α- and β-globins might indicate that a similar selective pressure has driven this divergence between primate species. To investigate whether bacterial heme-scavenging receptors are one such selective pressure, rapidly evolving sites were mapped onto a recently solved cocrystal structure between human hemoglobin and the IsdB protein from S. aureus (22). Notably, all four rapidly diverging hemoglobin residues are localized to the IsdB binding interface, in close proximity to the NEAT1 domain (Fig. 1C). Altogether, these findings indicate that primate globins have undergone rapid divergence at specific sites proximal to the binding interface of the S. aureus hemoglobin receptor IsdB.

10.1128/mBio.01524-18.1 DATA SET S1 Hemoglobin phylogenetic data. Download Data Set S1, XLSX file, 0.03 MB. Copyright © 2018 Choby et al. 2018 Choby et al. This content is distributed under the terms of the Creative Commons Attribution 4.0 International license.

### Primate hemoglobin variation dictates S. aureus binding and heme iron acquisition.

**FIG 2:**
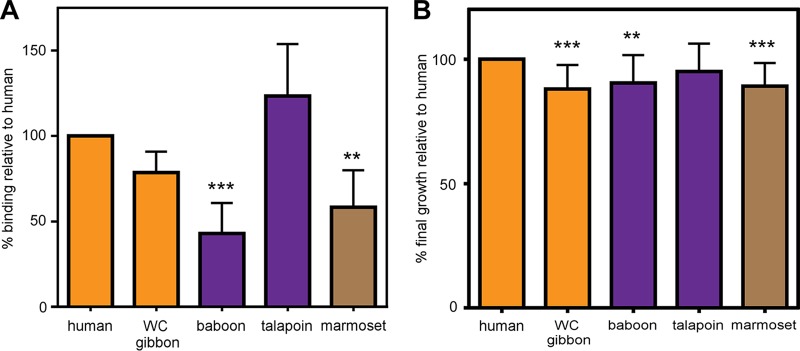
Primate hemoglobin variation dictates S. aureus binding and heme iron acquisition. (A) S. aureus binding of recombinant hemoglobin of various primate species. An iron-starved S. aureus wild type was incubated with purified recombinant hemoglobin from representative species across hominoid (orange), Old World monkey (purple), and New World monkeys (brown). Hemoglobin bound to the surface of S. aureus was eluted and analyzed by SDS-PAGE; relative hemoglobin abundance was measured by densitometry analysis (Image J) and compared to human hemoglobin for each replicate. (B) Growth of S. aureus in iron-depleted medium with 2.5 µg/ml of purified recombinant hemoglobin as the sole iron source. Shown is the final growth yield of S. aureus after 48 h. Growth of each replicate is compared to growth using human hemoglobin. Panel A shows the means from two independent experiments in biological triplicates, panel B shows the means from three independent experiments with 2 to 3 biological replicates, ± SEM; **, *P < *0.005; ***, *P < *0.0005 by two-way analyses of variance (ANOVA) with Sidak’s correction for multiple comparisons comparing transformed (percent value) data.

To assess how hemoglobin divergence among primates impacts recognition by S. aureus, recombinant hemoglobin from human, white-cheeked gibbon, baboon, talapoin, and marmoset sources were purified, providing a broad representation from our phylogenetic data set. An established biochemical assay was used to measure the binding of hemoglobin by S. aureus, in which S. aureus cells recognize recombinant human hemoglobin as well as hemoglobin purified from blood in an IsdB-dependent manner (see Fig. S1). S. aureus exhibited significantly reduced binding of baboon and marmoset hemoglobin to the cell surface (Fig. 2A and S2). It was noted that the binding patterns did not strictly match the predictions based on host phylogeny, suggesting discrete large-effect substitutions in hemoglobin may contribute disproportionately to the recognition by S. aureus. We next determined the ability of primate hemoglobins to support growth of S. aureus as the sole iron source. Consistent with the whole-cell binding data, hemoglobins that were bound by S. aureus with low affinity were unable to support optimal bacterial growth, indicating that the capability to bind hemoglobin is a measure of the ability to utilize hemoglobin as an iron source (Fig. 2B and S3). Altogether, these results demonstrate that variation among primate globins dictates bacterial hemoglobin capture and heme-dependent growth.

10.1128/mBio.01524-18.2 FIG S1 Whole-cell Hb binding assay enables IsdB-dependent species-specific quantification of bound recombinant Hb. A representative silver-stained SDS-PAGE gel used to assess binding of hemoglobin (Hb) to the surface of S. aureus; 100 ng of purified recombinant Hb was loaded (lane 2) to demonstrate apparent molecular weight in this gel, and the 15-kDa marker is marked, with an arrow indicating hemoglobin. The apparent hemoglobin band is specific to Hb, as it is not visible when S. aureus is incubated with PBS alone (lane 3). S. aureus binds human hemoglobin purified recombinantly from E. coli (lane 4) as well as Hb purified from blood (lane 5). Binding of Hb purified from mouse blood (lane 6) is significantly diminished, as first reported in reference 13. Binding of Hb is dependent on IsdB, as demonstrated in lanes 7 to 9, where the same hemoglobins were incubated with S. aureus lacking *isdB*. An approximately equivalent amount of S. aureus was used, as demonstrated by equal loading of non-Hb specific bands across the top of the gel. Download FIG S1, TIF file, 1.1 MB. Copyright © 2018 Choby et al. 2018 Choby et al. This content is distributed under the terms of the Creative Commons Attribution 4.0 International license.

10.1128/mBio.01524-18.3 FIG S2 Representative silver-stained SDS-PAGE gel of hemoglobin binding assay shown in Fig. 2. Each gel represents a biological replicate from independent binding assays. Molecular weight markers are indicated, with arrow showing hemoglobin band. Download FIG S2, TIF file, 1.9 MB. Copyright © 2018 Choby et al. 2018 Choby et al. This content is distributed under the terms of the Creative Commons Attribution 4.0 International license.

10.1128/mBio.01524-18.4 FIG S3 Growth of S. aureus using primate hemoglobin as the sole iron source. Graphed are the OD_600_ values at 24 and 48 h postinoculation with S. aureus wild type in iron-depleted medium containing hemoglobin of various primate species. Each dot is a single biological replicate from three independent experiments, with one replicate missing for talapoin. Download FIG S3, TIF file, 1.3 MB. Copyright © 2018 Choby et al. 2018 Choby et al. This content is distributed under the terms of the Creative Commons Attribution 4.0 International license.

### Species-specific diversity in α-globin restricts heme scavenging by S. aureus.

**FIG 3:**
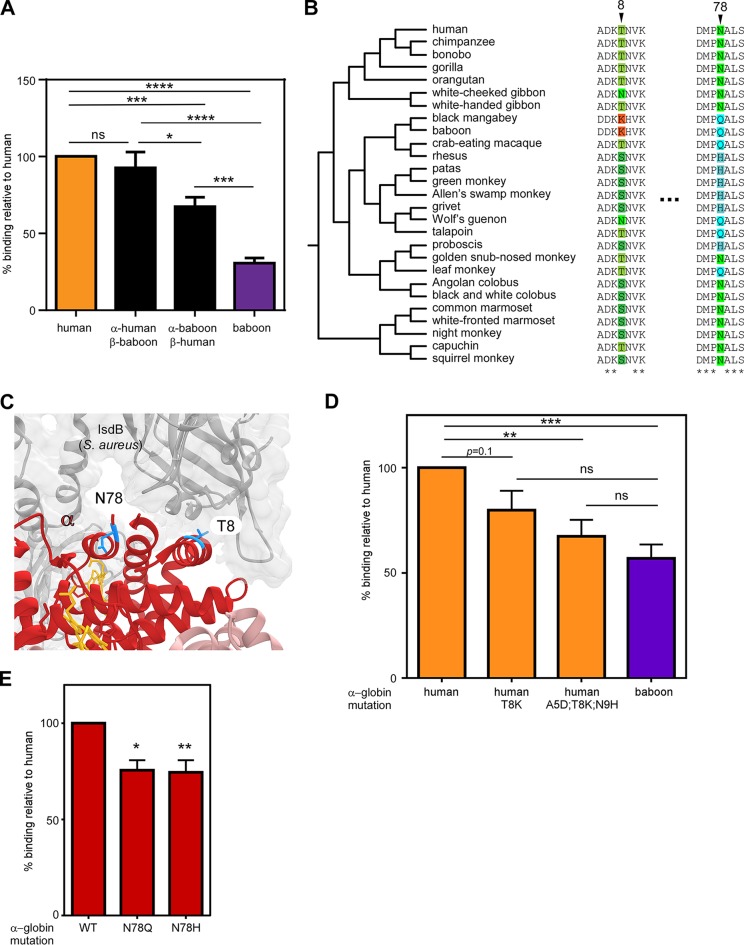
Species-specific diversity in α-globin restricts heme scavenging by S. aureus. (A). An iron-starved S. aureus wild type was incubated with purified recombinant hemoglobin, and bound hemoglobin was quantified. (B) Species phylogenies and sequence alignments surrounding positions exhibiting signatures of positive selection in α-globin. (C) Residues 8 and 78 of human α-globin (red) interact closely with IsdB (gray) (PDB 5VMM). (D) An iron-starved S. aureus wild type was incubated with purified recombinant hemoglobin, including mutagenized human hemoglobin, and bound hemoglobin was quantified. (E) An iron-starved S. aureus wild type was incubated with purified recombinant hemoglobin, including mutagenized human hemoglobin, and bound hemoglobin was quantified. Panel A shows the means from 3 independent experiments with 2 to 3 biological replicates, panel D shows the means from 6 independent experiments with 2 to 3 biological replicates, and panel E shows the means from 2 independent experiments with 3 biological replicates ± SEM; ns, no significance; *, *P < *0.05; **, *P < *0.005; ***, *P < *0.0005 by two-way ANOVA with Sidak’s correction for multiple comparisons comparing transformed (percent value) data.

The identification of rapidly evolving sites at the IsdB binding interface in both α-globin and β-globin suggests that both globin subunits contribute to S. aureus species-specific hemoglobin capture. We therefore exploited the enhanced binding of human hemoglobin relative to that from baboon to examine the role of each globin subunit in this biochemical interaction. The ability of S. aureus to bind chimeric hemoglobins was measured, which revealed that both globins contribute to species specificity (Fig. 3A and S4A), as chimeras containing either human α- or β-globin were bound more effectively than baboon hemoglobin. However, α-globin appears to have a greater effect on human-specific capture, as the α-human β-baboon chimera bound significantly better than α-baboon β-human. Focusing on phylogenetic variation at the protein binding interface, α-globin T8 and N78 were found to both be proximal to the NEAT1 domain of IsdB (Fig. 3B and C). Mutagenesis of the N-terminal alpha helix of human α-globin revealed that substituting the Thr residue of human α-globin with the Lys residue of baboon α-globin at position 8 reduced binding by S. aureus (Fig. 3D and S4B and C). Additionally, replacing A5D, T8K, and N9H in human α-globin, which converts this seven-amino-acid region (Fig. 3B), with those of baboon α-globin leaves S. aureus binding nearly indistinguishable from that of baboon hemoglobin. These results demonstrate that the N-terminal helix of α-globin makes a major contribution to human-specific hemoglobin recognition by S. aureus. Next, the relative importance of the rapidly evolving N78 residue in α-globin was assessed, which lies N terminal to the sixth alpha helix (Fig. 3B). The substitution of N78 for a glutamine (present in baboon, talapoin, and other Old World primates) or a histidine reduced the binding of human hemoglobin (Fig. 3E and S4D). Thus, substitutions at multiple residues in α-globin that exhibit signatures of repeated positive selection are sufficient to disrupt the ability of S. aureus to recognize human hemoglobin.

10.1128/mBio.01524-18.5 FIG S4 Representative silver-stained SDS-PAGE gel of hemoglobin binding assay shown in Fig. 3. (A) Corresponds to Fig. 3A, (B and C) correspond to Fig. 3D, where panel C shows results of experiments using an OD_600_ unit of 1 instead of 2. (D) Corresponds to Fig. 3E. Each gel represents a biological replicate from independent binding assays. Molecular weight markers are indicated, with arrow showing hemoglobin band. Download FIG S4, TIF file, 2.3 MB. Copyright © 2018 Choby et al. 2018 Choby et al. This content is distributed under the terms of the Creative Commons Attribution 4.0 International license.

### β-Globin divergence contributes to S. aureus hemoglobin binding.

**FIG 4:**
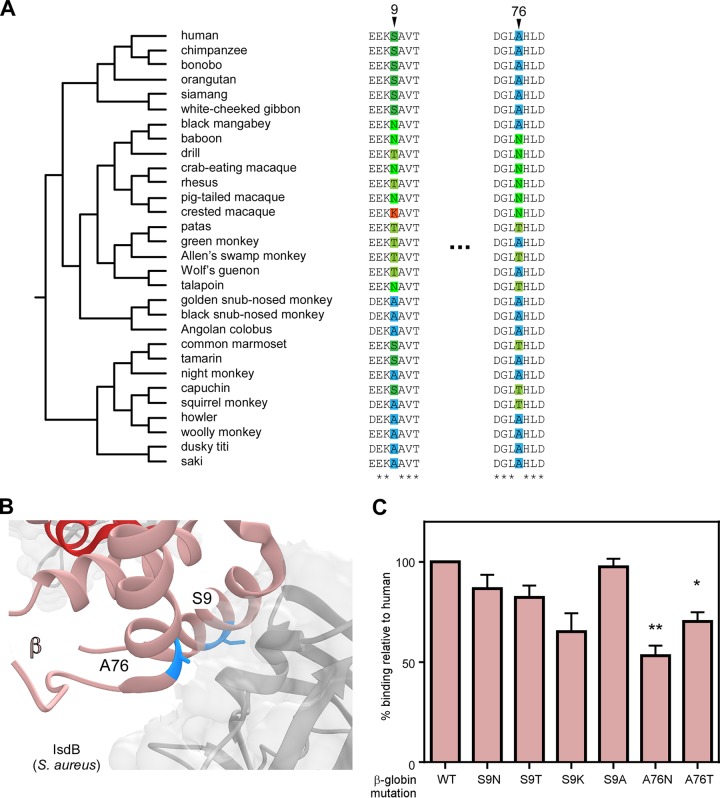
β-Globin divergence contributes to S. aureus hemoglobin binding. (A) Species phylogenies and sequence alignments surrounding positions exhibiting signatures of positive selection in β-globin. (B) Residues 9 and 76 of human β-globin (salmon) interact closely with IsdB (gray) (PDB 5VMM). (C) An iron-starved S. aureus wild type was incubated with purified recombinant human hemoglobin or variants of human hemoglobin encoding variants in β-globin, and bound hemoglobin was quantified. The means from four independent experiments with 3 biological replicates ± SEM are shown; *, *P < *0.05; **, *P < *0.005 by two-way ANOVA with Sidak’s correction for multiple comparisons comparing transformed (percent value) data.

S. aureus was capable of binding the baboon α-globin human β-globin chimeric hemoglobin with higher affinity than baboon hemoglobin (Fig. 3A), signifying that β-globin also contributes to S. aureus species-specific hemoglobin capture. Therefore, the contribution of rapidly evolving residues in β-globin to this binding interaction was investigated (Fig. 4A). Both S9 and A76 interact closely with the NEAT1 domain of IsdB (Fig. 4B). The effect of replacing the human β-globin S9 and A76 with residues found in other primate species analyzed in this work was systematically tested, which revealed that A76 is particularly important for binding by S. aureus (Fig. 4C and S5). Notably, baboon and human β-globins differ at both positions 9 and 76, suggesting that these residues contribute to the inability of IsdB to bind baboon hemoglobin. These differences might also explain the different binding affinities between human hemoglobin and the human α-globin baboon β-globin chimera, observed in Fig. 3A. As for α-globin, no single residue substitution improved binding by S. aureus IsdB, consistent with the hypothesis that IsdB has specifically adapted to bind human hemoglobin. Taken together with earlier data, residues at the IsdB interface of both α-globin and β-globin contribute to the recognition of hemoglobin by S. aureus. This is consistent with the NEAT1 domain of multiple IsdB monomers engaging in hemoglobin capture by binding both α- and β-globins, as observed in the reported cocrystal structure (22).

10.1128/mBio.01524-18.6 FIG S5 Representative silver-stained SDS-PAGE gel of hemoglobin binding assay shown in Fig. 4. Each gel represents a biological replicate from independent binding assays. Molecular weight markers are indicated, with arrow showing hemoglobin band. Download FIG S5, TIF file, 2.8 MB. Copyright © 2018 Choby et al. 2018 Choby et al. This content is distributed under the terms of the Creative Commons Attribution 4.0 International license.

### IsdB diversity among related staphylococcal strains impacts primate-specific hemoglobin capture.

**FIG 5:**
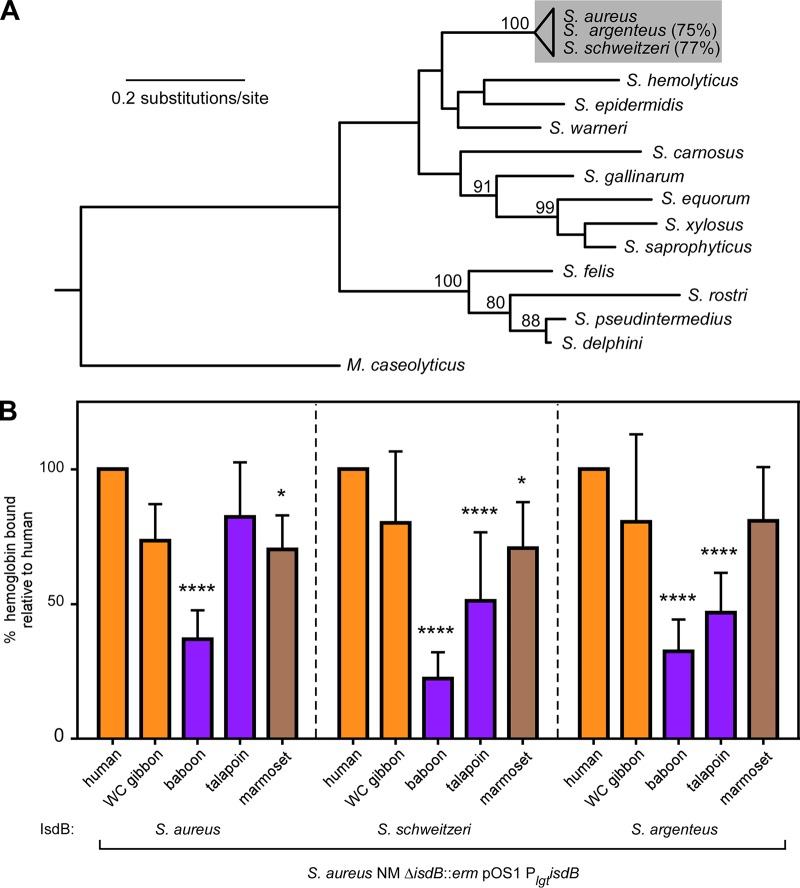
IsdB diversity among related staphylococcal strains impacts primate-specific hemoglobin capture. (A) Maximum likelihood phylogeny of the DNA gyrase A protein from representative staphylococci generated using PhyML. M. caseolyticus was included as an outgroup. The similarity of IsdB in S. argenteus and S. schweitzeri relative to S. aureus is shown on the right. Bootstrap values above 80 are indicated. (B) S. aureus lacking native *isdB* but harboring constitutively expressed plasmid-borne *isdB* variants were incubated with purified recombinant hemoglobin and from hominoid (orange), Old World monkey (purple), and New World monkeys (brown) and bound hemoglobin was quantified. The means from three independent experiments with 3 biological replicates ± SEM are shown; *, *P < *0.05; ****, *P < *0.0001 by two-way ANOVA with Sidak’s correction for multiple comparisons, comparing transformed (percent value) data.

Given the observed differences in S. aureus binding between diverse primate hemoglobins, we considered how genetic variation in IsdB might impact this interaction. The IsdB NEAT1 subdomain Q162R-S170T is critical for hemoglobin recognition and is completely conserved among more than three thousand S. aureus clinical isolates (11). Therefore, IsdB variation among congeneric Staphylococcus argenteus and Staphylococcus schweitzeri was assessed. These recently diverged taxa (Fig. 5A) are both primate associated and, unlike most other staphylococci, harbor IsdB. We measured the ability of IsdB from S. argenteus and S. schweitzeri to bind hemoglobin by expressing them ectopically in S. aureus lacking the native *isdB* gene. Consistent with their overall high sequence identity, S. schweitzeri and S. argenteus IsdB bound primate hemoglobin with a similar pattern of species preference as S. aureus (Fig. 5B and S6). However, both the IsdB of S. schweitzeri and S. argenteus displayed reduced binding of talapoin hemoglobin, and S. argenteus IsdB did not bind marmoset hemoglobin significantly less than human hemoglobin. These data indicate that the variation among IsdB sequences impacts species-specific hemoglobin capture.

10.1128/mBio.01524-18.7 FIG S6 Representative silver-stained SDS-PAGE gel of hemoglobin binding assay shown in Fig. 5. Each gel represents a biological replicate from independent binding assays. Molecular weight markers are indicated, with arrow showing hemoglobin band. Download FIG S6, TIF file, 2 MB. Copyright © 2018 Choby et al. 2018 Choby et al. This content is distributed under the terms of the Creative Commons Attribution 4.0 International license.

### IsdB NEAT1 domain diversity among staphylococci modulates human hemoglobin recognition.

**FIG 6:**
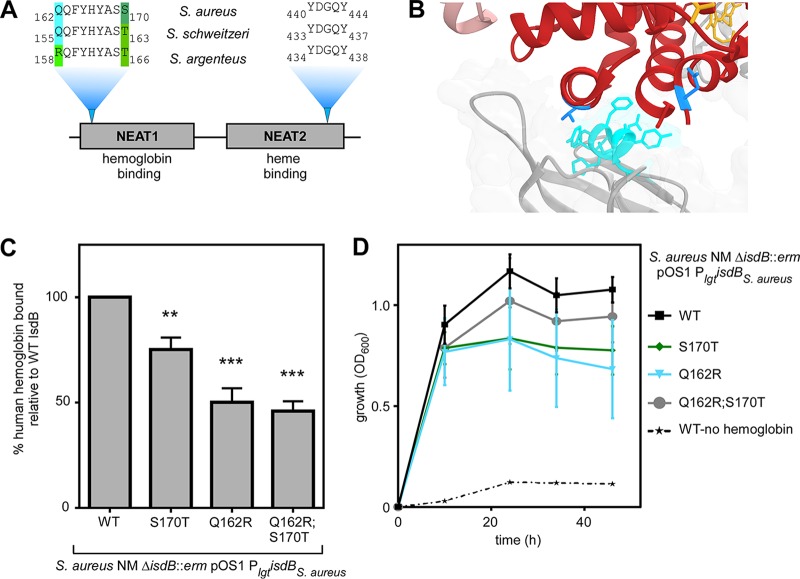
IsdB NEAT1 domain diversity among staphylococci modulates human hemoglobin recognition. (A) An alignment of the NEAT1 subdomain critical for hemoglobin binding shows variation among staphylococcal IsdB, while no variation was observed for the NEAT2 subdomain required for heme binding. (B) The Q162 to S170 subdomain of NEAT1 (cyan) is proximal to helices containing T8 and N78 of α-globin (red). (C) S. aureus lacking native *isdB* but harboring constitutively expressed plasmid-borne S. aureus *isdB* variants was incubated with purified recombinant human hemoglobin, and bound hemoglobin was quantified. (D) The growth of S. aureus lacking native *isdB* but harboring constitutively expressed plasmid-borne S. aureus *isdB* variants using hemoglobin as the sole iron source was monitored over time. Panel C shows, the means from three independent experiments with 3 biological replicates ± SEM; **, *P < *0.005; ***, *P < *0.0005 by two-way ANOVA with Sidak’s correction for multiple comparisons, comparing transformed (percent value) data. Panel D shows the results of two independent experiments with six biological replicates each ± standard deviations.

A closer examination of the Q162R-S170T region of IsdB NEAT1 revealed variation between related staphylococci but no variation in the critical heme-binding region of NEAT2 (Fig. 6A). This region of NEAT1 closely interacts with the N-terminal helices of either α-globin or β-globin, in close proximity to both discrete sites bearing signatures of adaptive evolution in α-globin and β-globin (Fig. 6B). To determine the functional consequences of variation in this NEAT1 domain, Q162 and S170T were mutagenized in S. aureus IsdB to mimic the sequence of S. schweitzeri and S. argenteus. These residues are not expected to disrupt IsdB tertiary structure, as they already exist in related IsdB proteins. Variations at both of these positions reduced the affinity for human hemoglobin, showing that in the context of S. aureus IsdB, Q162 and S170 are required for high-affinity hemoglobin binding (Fig. 6C and S7). Additionally, mutagenized IsdB failed to fully support the growth of S. aureus using human hemoglobin as the sole iron source (Fig. 6D), which supports the conclusion that this NEAT1 subdomain of S. aureus has evolved for optimal binding and utilization of human hemoglobin.

10.1128/mBio.01524-18.8 FIG S7 Representative silver-stained SDS-PAGE gel of hemoglobin binding assay shown in Fig. 6. Each gel represents a biological replicate from independent binding assays. Molecular weight markers are indicated, with arrow showing hemoglobin band. Download FIG S7, TIF file, 1.4 MB. Copyright © 2018 Choby et al. 2018 Choby et al. This content is distributed under the terms of the Creative Commons Attribution 4.0 International license.

## DISCUSSION

In this work, we report that recurrent positive selection acting on primate α- and β-globin proteins restricts hemoglobin binding and nutrient acquisition by pathogenic S. aureus. Estimations of divergence in the *Staphylococcus* genus have been lacking; however, the Kloos hypothesis (41) contends that staphylococci have coevolved with their mammalian hosts over long evolutionary timescales. In support of this concept, primate specificity among staphylococci has been reported, including S. aureus, S. epidermidis, and S. warneri, as well as avian (S. gallinarum), equine (S. equorum), and other taxa. Indeed, it has been proposed that the canine-associated S. pseudintermedius diverged from S. aureus simultaneously with the divergence of Primate and Carnivora orders (42). Most staphylococci are commensal organisms, while S. aureus is uniquely adapted to infect deep tissue and cause disease. As such, the IsdB system is only harbored by S. aureus and closely related primate-associated staphylococci. By narrowing our analysis of hemoglobin evolution to primates, we were thus able to assess specific biological features of primate-associated staphylococci.

An outstanding question in the study of S. aureus evolution has been determining the selective pressures responsible for human-specific virulence factors. S. aureus asymptomatically colonizes the anterior nares of approximately one-third of the human population yet is capable of causing a wide range of invasive diseases. While some bacterial colonization factors have been implicated in the pathogenesis, many virulence factors have evolved highly specific targets that are not obviously involved in nasal colonization (43). As such, we cannot definitively conclude that IsdB evolution has been driven by selection during invasive disease. It is also likely that variation across IsdB sequences of S. aureus, S. argenteus, and S. schweitzeri may be the results of antigenic variation to evade the immune system. By focusing on the hemoglobin binding pocket of IsdB, we have been able to pinpoint critical variations for hemoglobin specificity.

Our phylogenetic analyses revealed strikingly parallel signatures of positive selection between the α- and β-globin genes across primates. In particular, rapidly evolving sites in the α- and β-globin A-helices are predicted to be homologous on the basis of the predicted protein alignments. Our results suggest that these correlations reflect selection in response to NEAT domain-containing bacterial receptors like IsdB with conserved globin-binding sites. A well-established body of literature has shown that other selective pressures play important roles in the patterns of hemoglobin polymorphism and divergence across vertebrates, including the adaptation to high altitude and malaria resistance (34, 38). It is therefore possible that the signatures of selection detected in our study were driven by pressures other than nutritional immunity. Nonetheless, our empirical results demonstrate that variation in hemoglobins at discrete sites has important functional consequences for bacterial iron acquisition.

Previous studies have illustrated how mutations in hemoglobin coding or regulatory regions can have highly deleterious effects on heme binding, oxygen affinity, and protein stability (44, 45). In addition to aforementioned sickle-cell alleles, dozens of hemoglobin mutations in humans have been reported that contribute to genetic disease, including anemia and thalassemia (46). Thus, despite identifying particular sites that are highly divergent among primates, much of the globin gene content is constrained due to purifying selection. In future work, it would be useful to determine how variation among primate globins impacts other biochemical functions, including heme binding and oxygen affinity. Such insights might improve our fundamental understanding of hemoglobin biology and the mechanisms underlying human hemoglobinopathies.

In conclusion, this work illustrates how rapid site-specific hemoglobin variation restricts heme acquisition by the prominent human pathogen S. aureus. These findings provide a fundamental new perspective on vertebrate globin evolution, highlighting nutritional immunity as a selective pressure that might strongly impact divergence and natural selection. Future studies will assist in illuminating how these combinations of adaptive mutations contribute to hemoglobin function and host physiology. An understanding of the genetic and molecular determinants of bacterial pathogenicity is critical for developing new antimicrobial treatment strategies, particularly as major pathogens such as S. aureus continue to develop resistance to existing antibiotics. Combining comparative genetics with molecular experimentation in turn provides not only a historical perspective of host-microbe evolutionary conflict but also mechanistic insights on modern human infectious disease.

## MATERIALS AND METHODS

### Bacterial strains.

The bacterial strains and plasmids used in this study are listed in Table 1. For Escherichia coli strains, LB agar and broth (Fisher, Hampton, NH) were routinely used, and growth was at 37°C. For the selection of pHUG21, 12.5 µg/ml of carbenicillin (Fisher) was used, for the selection of pHb0.0, 5 µg/ml of tetracycline hydrochloride (Alfa Aesar, Haverhill, MA) was used, and for the selection of pOS1 P*_lgt_*, 50 µg/ml of carbenicillin was used. *Staphylococcus* strains were grown at 37°C using tryptic soy agar and broth (Fisher), except where noted throughout. For the selection of pOS1 P*_lgt_*, 10 µg/ml chloramphenicol (Fisher) was used except where noted. The strains were streaked onto agar from stocks stored at −80°C 2 days prior to each experiment.

**TABLE 1:** Strains and plasmids

Strain or plasmid	Source and/or reference
Strains	
Escherichia coli DH5α	Laboratory stock, Thermo Fisher
Escherichia coli BL21(DE3) pHUG21	Douglas Henderson (University of Texas of the Permian Basin); 52
Staphylococcus aureus RN4220	53
S. aureus Newman	Laboratory stock; 54
S. aureus Newman Δ*isdB::erm*	7
S. argenteus MSHR1132	DSMZ
S. schweitzeri FSA084	DSMZ
Plasmids	Source
pHb0.0-human	John Olson (Rice University); 52
pHb0.0-white-cheeked gibbon	This paper
pHb0.0-baboon	This paper
pHb0.0-talapoin	This paper
pHb0.0-marmoset	This paper
pHb0.0-alpha_human_beta_baboon_	This paper
pHb0.0-alpha_baboon_beta_human_	This paper
pHb0.0-human αT8K	This paper
pHb0.0-human αA5D;T8K;N9H	This paper
pHb0.0-human αN78Q	This paper
pHb0.0-human αN78H	This paper
pHb0.0-human βS9N	This paper
pHb0.0-human βS9T	This paper
pHb0.0-human βS9K	This paper
pHb0.0-human βS9A	This paper
pHb0.0-human βA76N	This paper
pHb0.0-human βA76T	This paper
pOS1 P*_lgt_*	55
pOS1 P*_lgt_isdB_aureus_*	This paper
pOS1 P*_lgt_isdB_schweitzeri_*	This paper
pOS1 P*_lgt_isdB_argenteus_*	This paper
pOS1 P*_lgt_isdB_aureus_* Q162R	This paper
pOS1 P*_lgt_isdB_aureus_* S170T	This paper
pOS1 P*_lgt_isdB_aureus_* Q162R;S170T	This paper

### Hemoglobin cloning and genetic manipulation.

We compiled a subset of α- and β-globin sequences from GenBank, as well as cloned α-globin orthologs from cDNA derived from primate cell lines and β-globin orthologs from primate genomic DNA. Hemoglobin gene sequences have been deposited in GenBank (accession numbers MH382883 to MH382906). Hemoglobin gene sequences obtained from GenBank include those for olive baboon, bonobo, white-headed capuchin, chimpanzee, Angolan colobus, northern white-cheeked gibbon, green monkey, human, drill, crab-eating macaque, common marmoset, Sumatran orangutan, rhesus macaque, black snub-nosed monkey, golden snub-nosed monkey, and squirrel monkey. Primate cell lines were purchased from the Coriell Institute for Medical Research (Camden, NJ). The α-globin orthologs cloned from cDNA (with Coriell identifier [ID] numbers) are as follows: African green monkey (PR01193), black-and-white colobus (PR00240), white-handed gibbon (PR01131), Western lowland gorilla (AG05251), Francois’ leaf monkey (PR01099), black crested mangabey (PR01215), white-faced marmoset (PR00789), Nancy Ma’s night monkey (PR00627), patas monkey (AG06116), proboscis monkey, Allen’s swamp monkey (PR01231), talapoin (PR00716), and Wolf’s guenon (PR00486). The β-globin orthologs cloned from genomic DNA (with Coriell ID numbers) are as follows: crested macaque (PR01215), Bolivian red howler monkey (PR00708), pigtailed macaque, black-crested mangabey (PR01215), Nancy Ma’s night monkey(PR00627), patas monkey (AG06116), white-faced saki (PR00239), island siamang (PR00722), Allen’s swamp monkey (PR01231), talapoin (PR00716), Spix’s saddle-back tamarin (AG05313), dusky titi (PR00742), Wolf’s guenon (PR00486), and common woolly monkey (PR00525).

Primate hemoglobin cDNA was cloned into pHb0.0 using Gibson assembly (New England Biolabs [NEB], Ipswich, MA). In general, each α- and β-globin gene cDNA was amplified from the template (above) using Phusion 2× master mix (Thermo, Waltham, MA) with primers that also had homology to pHb0.0. All primers are listed in Table S1 in the supplemental material. Because of cDNA sequence homology, some primers were used for multiple species. pHb0.0-human was digested with PacI (NEB) and HindIII-HF (NEB), and the doubly digested vector was isolated by gel purification (Qiagen, Germantown, MD). PCR products were assembled with digested pHb0.0, transformed to DH5α, reisolated with a Mini-prep (Thermo), and confirmed by sequencing (GeneWiz, South Plainfield, NJ) with primers pHb0.0_for/pHb0.0_rev. Globin cDNA was amplified with the following primers for Gibson assembly: white-cheeked gibbon α-globin, primers AF327/328; white-cheeked gibbon β-globin, primers AF329/330; baboon α-globin, primers AF331/332; baboon β-globin, primers AF329/330; talapoin α-globin, primers AF327/328; talapoin β-globin, primers AF329/330; marmoset α-globin, primers AF333/334; and marmoset β-globin, primers AF329/335.

10.1128/mBio.01524-18.10 TABLE S1 Oligonucleotides used in this study. Download Table S1, DOCX file, 0.01 MB. Copyright © 2018 Choby et al. 2018 Choby et al. This content is distributed under the terms of the Creative Commons Attribution 4.0 International license.

Chimeric hemoglobins were prepared by subcloning the α-globins. To enable digestion with XbaI (NEB) (which is sensitive to *dam* methylation) pHb0.0-human and pHb0.0-baboon were transformed and reisolated from E. coli K1077 (Δ*dam* Δ*dcm*). The α-globin from each plasmid was excised by digestion with XbaI (NEB) and PacI (NEB), and the α-globin and doubly digested pHb0.0 containing β-globin were separately isolated by gel purification (Qiagen). Human α-globin was ligated into pHb0.0 containing baboon β-globin and baboon α-globin was ligated (T4 ligase; NEB) into pHb0.0 containing human β-globin. The chimeras were confirmed by sequencing (GeneWiz) with primers pHb0.0_for/pHb0.0_rev.pHb0.0-human was mutagenized using a QuikChange site-directed mutagenesis kit (Agilent, Santa Clara, CA) to create changes in the α-gene: T8K (primers AF289/290), T8K/N9H (primers JC112/113; using pHb0.0-human αT8K as the template), A5D/T8K/N9H (primers JC114/115; using pHb0.0-human αT8K;N9H as the template), N78Q (primers AF291/292), and N78H (primers AF293/294). Changes in the human β-gene were as follows: S9N (primers AF303/304), S9T (primers AF307/308), S9A (primers AF309/310), S9K (primers AF305/306), A76N (primers AF313/314), and A76T (primers AF311/312).

### Phylogenetic analyses.

Hemoglobin DNA sequence alignments were performed using MUSCLE. Input phylogenies were based upon supported species relationships (47) as well as maximum likelihood gene phylogenies generated using PhyML with SPR topology search and 1,000 bootstraps for branch support (48). Tests for positive selection were performed using codeml from the PAML software package with the F3X4 codon frequency model. Likelihood ratio tests (LRTs) were performed by comparing pairs of site-specific models (NS sites): M1 (neutral) with M2 (selection), M7 (neutral, beta distribution of *dN/dS* < 1) with M8 (selection, beta distribution, *dN/dS* > 1 allowed). Additional tests which also account for synonymous rate variation and recombination, including FUBAR, FEL, and MEME, were performed using the HyPhy software package via the Datamonkey server (49, 50). Sites under positive selection were mapped onto three-dimensional molecular structures by using Chimera (51) (http://www.cgl.ucsf.edu/chimera/).

The staphylococcal DNA gyrase gene tree was generated using PhyML with 1,000 bootstraps as described above. Macrococcus caseolyticus DNA gyrase was included as an outgroup. The similarity of IsdB sequences in S. argenteus and S. schweitzeri relative to that in S. aureus is shown in Fig. 5.

### Recombinant purification of hemoglobin.

Hemoglobin expression strains [BL21(DE3) pHUG21 pHb0.0] were streaked onto LB agar containing 12.5 µg/ml carbenicillin and 5 µg/ml tetracycline hydrochloride. pHb0.0 harbors both α- and β-globin genes, and proper folding and tetramerization require sufficient intracellular heme. Therefore, pHUG21, which encodes a heme uptake system, is coexpressed, and hemin is supplemented in the medium (52). Single colonies were inoculated in 5 ml of LB broth supplemented with 12.5 µg/ml carbenicillin and 5 µg/ml tetracycline hydrochloride and grown for 14 h at 37°C with shaking. This culture was used for inoculating 1:500 into 1.5 liters of LB with 12.5 µg/ml carbenicillin, 5 µg/ml tetracycline hydrochloride, 100 µM hemin (prepared fresh at 10 mM in 0.1 M NaOH; Sigma, St. Louis, MO), and 50 µg/ml of the iron chelator ethylenediamine-di(*o*-hydroxyphenylacetic acid (EDDHA, solid added directly to medium; LGC Standards, Teddington, UK; ) in a 2.8-liter Fernbach flask. Cultures were grown at 37°C until the optical density at 600 nm (OD_600_) reached 0.6 to 0.8. The expression of hemoglobin was induced with 40 µg/ml IPTG (isopropyl-β-isopropyl-β-d-thiogalactopyranoside-thiogalactopyranoside; RPI, Mount Prospect, IL). At 16 h postinduction at 37°C, cells were collected by centrifugation. The cell pellet was resuspended in 20 ml phosphate-buffered saline (PBS) containing 10 mM imidazole (Fisher), 5 mM MgCl_2_ (Sigma), 1 Roche protease inhibitor tablet (Fisher), approximately 1 mg/ml of lysozyme (Thermo), and 100 µg/ml DNase from bovine pancreas (Sigma). The cell pellet resuspended with rocking for 20 min at room temperature after incubating on ice for 20 min. The cells were lysed using an Emulsiflex (Avestin, Ottawa, CA), and then the cell lysate was clarified by ultracentrifugation (60 min at 17,000 × g). The cell lysate was applied to a 3 ml of nickel-nitrilotriacetic acid (Ni-NTA) resin (Qiagen) in a gravity column, to which hemoglobin binds, and washed with 50 ml PBS containing 10 mM imidazole. Hemoglobin was eluted with 6 ml PBS containing 500 mM imidazole, with the first 1 ml of eluate discarded. The hemoglobin-containing eluate was dialyzed twice sequentially in PBS at 4°C. Purified hemoglobin was filter sterilized with a 0.45-μm filter and stored in aliquots in liquid nitrogen. The hemoglobin concentration was measured with Drabkin’s reagent (Sigma) using human hemoglobin as a standard, ranging from 2 to 6 mg/ml. The relative purity was assessed using SDS-PAGE before use in experiments, as shown in Fig. S8.

10.1128/mBio.01524-18.9 FIG S8 SDS-PAGE of representative purified hemoglobins used throughout the manuscript. One microgram of hemoglobin was subjected to SDS-PAGE (4% to 20% gradient) and stained with Imperial protein stain. Molecular weight markers are indicated, with arrow showing hemoglobin band. High-molecular-weight bands may be contamination or unresolved dimers and tetramers. Download FIG S8, TIF file, 2 MB. Copyright © 2018 Choby et al. 2018 Choby et al. This content is distributed under the terms of the Creative Commons Attribution 4.0 International license.

### Whole-cell hemoglobin binding assay.

S. aureus strains were streaked on tryptic soy agar (containing 10 µg/ml chloramphenicol for strains carrying plasmids) and grown at 37°C for 24 h. Single colonies were used to inoculate 3 ml of RPMI containing 1% Casamino Acids and 0.5 mM 2,2′-dipyridyl (Acros/Fisher) to induce the expression of chromosomal *isdB* or 10 µg/ml chloramphenicol (for strains carrying plasmids with constitutive *isdB* expression). After 14 to 16 h of growth at 37°C with shaking, 2 OD_600_ units (except for experiments shown in part in Fig. 3D and S4C, where 1 OD_600_ unit was used) were collected by centrifugation in a 1.5-ml Eppendorf tube. The cell pellet was resuspended with 1 ml PBS or PBS containing recombinant hemoglobin. Then, 10 µg/ml (chromosomal *isdB*) or 2.5 µg/ml (plasmid-borne *isdB*) of hemoglobin was used. The cells were incubated with hemoglobin or PBS for 30 min at 37°C with shaking, and then the cells were collected by centrifugation at 4°C at 8,000 × *g*. Cells were washed thrice by pipetting with 1 ml ice-cold PBS and centrifuging at 4°C at 8,000 × *g*. After the final wash, the cells were resuspended in 30 µl 0.5 M Tris (pH 8.0; Fisher) containing 4% SDS (Fisher) and heated at 90°C for 5 min to remove surface-bound proteins. The cells were collected by centrifugation at 8,000 × *g*, and the eluate was added to 6× loading buffer and heated at 90°C for 5 min. The samples were subjected to 12% or 17.5% SDS-PAGE and silver stained (GE, Boston, MA). The gels were imaged using an Alpha Innotech Alpha Imager or Bio-Rad ChemiDoc MP imaging system. Quantification was performed by densitometric analysis with Image J (NIH) according to the software instructions and by quantifying the area under the peak that corresponds to the hemoglobin band, excluding background density. Because of the variation in stain intensity and quantity of nonspecific bands across gels, all comparisons were made within the same gel, and the relative density was calculated for each biological replicate within the same gel; the comparison was either to human hemoglobin or wild-type IsdB, depending on the assay. Additionally, PBS-only samples and S. aureus Δ*isdB*::*erm* were used to verify that hemoglobin binding in this assay is IsdB dependent (Fig. S4C, S5, and S8) as previously observed, and that recombinant human hemoglobin is bound equally as well as hemoglobin purified from human blood (Fig. S1) (11, 13).

### Growth with hemoglobin as sole iron source.

For hemoglobin variants (Fig. 2D and S3), S. aureus Newman WT was streaked onto tryptic soy agar and allowed to grow for 24 h at 37°C. A few colonies were used to inoculate 5 ml of RPMI (Corning, Corning, NY) supplemented with 1% Casamino Acids (Fisher) and 0.5 mM EDDHA (prepared fresh in ethanol). After growth to stationary phase at 37°C with shaking, approximately 16 h, 4 µl of culture was inoculated in 196 µl of medium in a 96-well plate, and the OD_600_ at 37°C with shaking was monitored over time using a BioTek plate reader. The medium was RPMI containing 1% Casamino Acids that had been depleted of cations with Chelex 100 (Sigma) according to the manufacturer’s instructions, filter sterilized, and supplemented with 25 μM ZnCl_2_, 25 μM MnCl_2_, 100 μM CaCl_2_, and 1 mM MgCl_2_ (all from Fisher) to restore noniron cations, 1.5 mM EDDHA to chelate any remaining free iron, and 2.5 µg/ml of recombinant purified hemoglobin as the sole iron source.

For IsdB variants (Fig. 6D), S. aureus strains were streaked onto tryptic soy agar containing 10 µg/ml chloramphenicol and allowed to grow for 24 h at 37°C. A single colony was resuspended in 120 µl RPMI containing 1 µM EDDHA (prepared fresh in 0.1 M NaOH) and 5 µg/ml chloramphenicol. Then, 100 µl was added to 2 ml of RPMI containing 1 µM EDDHA and 5 µg/ml chloramphenicol and grown at 37° with shaking in aeration tubes for 8 h. The OD_600_ was measured for each culture and normalized to 1, and 5 µl was used to inoculate 2 ml of RPMI containing 1 µM EDDHA, 5 µg/ml chloramphenicol, and 50 nM hemoglobin or no hemoglobin (for Δ*isdB* pOS1 P*_lgt_isdB*). Growth was monitored every 12 h by removing 50 µl of culture and adding to 150 µl PBS to measure the OD_600_ with path length correction in a BioTek plate reader, with the background subtracted from the corrected OD_600_ values. Growth using hemoglobin as a sole iron source in both assays is IsdB dependent (11, 13, 22).

### Cloning of *isdB*.

The full-length coding sequences of IsdB were amplified from genomic DNA using Phusion 2× high-fidelity master Mix (Thermo); cells were treated with 20 µg of lysostaphin (AMBI Products, Lawrence, NY), and DNA was isolated with a Wizard genomic DNA extraction kit (Promega, Madison, WI). S. aureus *isdB* (*NWMN_1040*) was amplified using primers JC343/344, S. schweitzeri *isdB* (*ERS140239_01018*) using primers JC218/219, and S. argenteus *isdB* (*SAMSHR1132_09750*) using primers JC216/217. Each primer pair included homology to pOS1 P*_lgt_* digested with NdeI and BamHI-HF (NEB), and PCR products were ligated to pOS1 P*_lgt_* with Hi-Fi assembly (NEB), transformed into E. coli DH5α, and reisolated with a Mini-prep (Thermo). All plasmids were sequence confirmed by Sanger sequencing (GeneWiz). Plasmids were transformed into RN4220 by electroporation, reisolated, and transformed into S. aureus Newman Δ*isdB*::*erm* by electroporation.

### S. aureus IsdB site-directed mutagenesis.

pOS1 P*_lgt_isdB_aureus_* was subjected to site-directed mutagenesis by PCR with a Q5 site-directed mutagenesis kit (NEB). Primer pairs with desired mutation were used to create Q162R (primers JC315/316), S170T (primers 317/318) and Q162R;S170T (primers JC319/320). PCR products were transformed to DH5α. Plasmids were isolated and subjected to Sanger sequencing with primers JC228/229 (GeneWiz) to identify successful incorporation of the desired mutation. Plasmids were transformed into RN4220 by electroporation, reisolated, and transformed into S. aureus Newman Δ*isdB*::*erm* by electroporation.

### Quantification and statistical analysis.

Specific statistical details for each experiment can be found in the corresponding figure legends. Data analysis and statistical tests were performed in Prism 6 (GraphPad).

## ADDENDUM IN PROOF

While our article was in press, a complementary article was published by A. Mozzi et al. (Front Immunol 9:2086, 2018, https://doi.org/10.3389/fimmu.2018.02086).
