# Supplementary figures and images for: Molecular Basis for the Evolution of Species-Specific Hemoglobin Capture by Staphylococcus aureus
Source: mBio. 2018 Nov 20;9(6):e01524-18. doi: 10.1128/mBio.01524-18 (PMC6247092; doi:10.1128/mBio.01524-18)

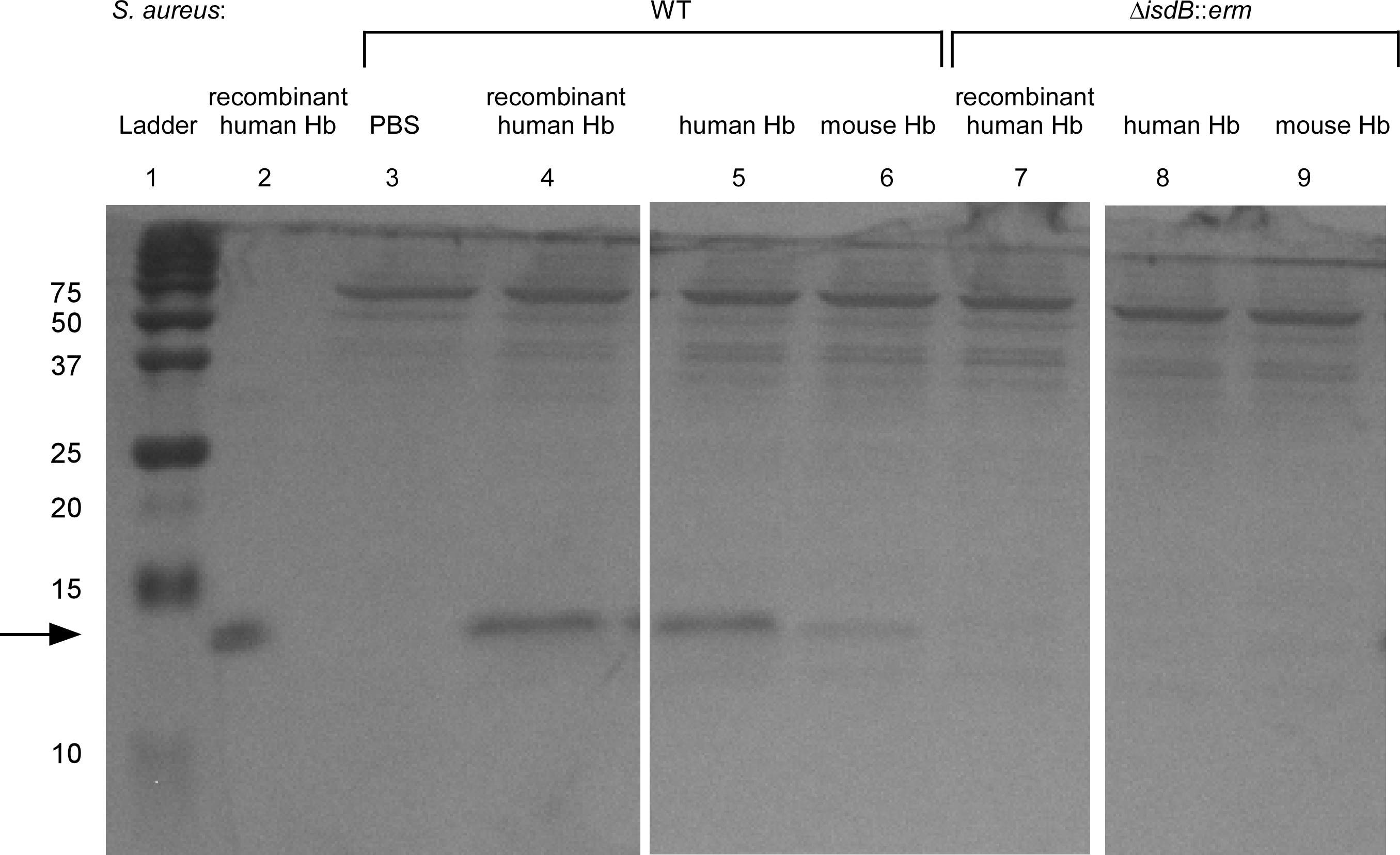

Supplement: FIG S1 [file mbo006184177sf1.tif]

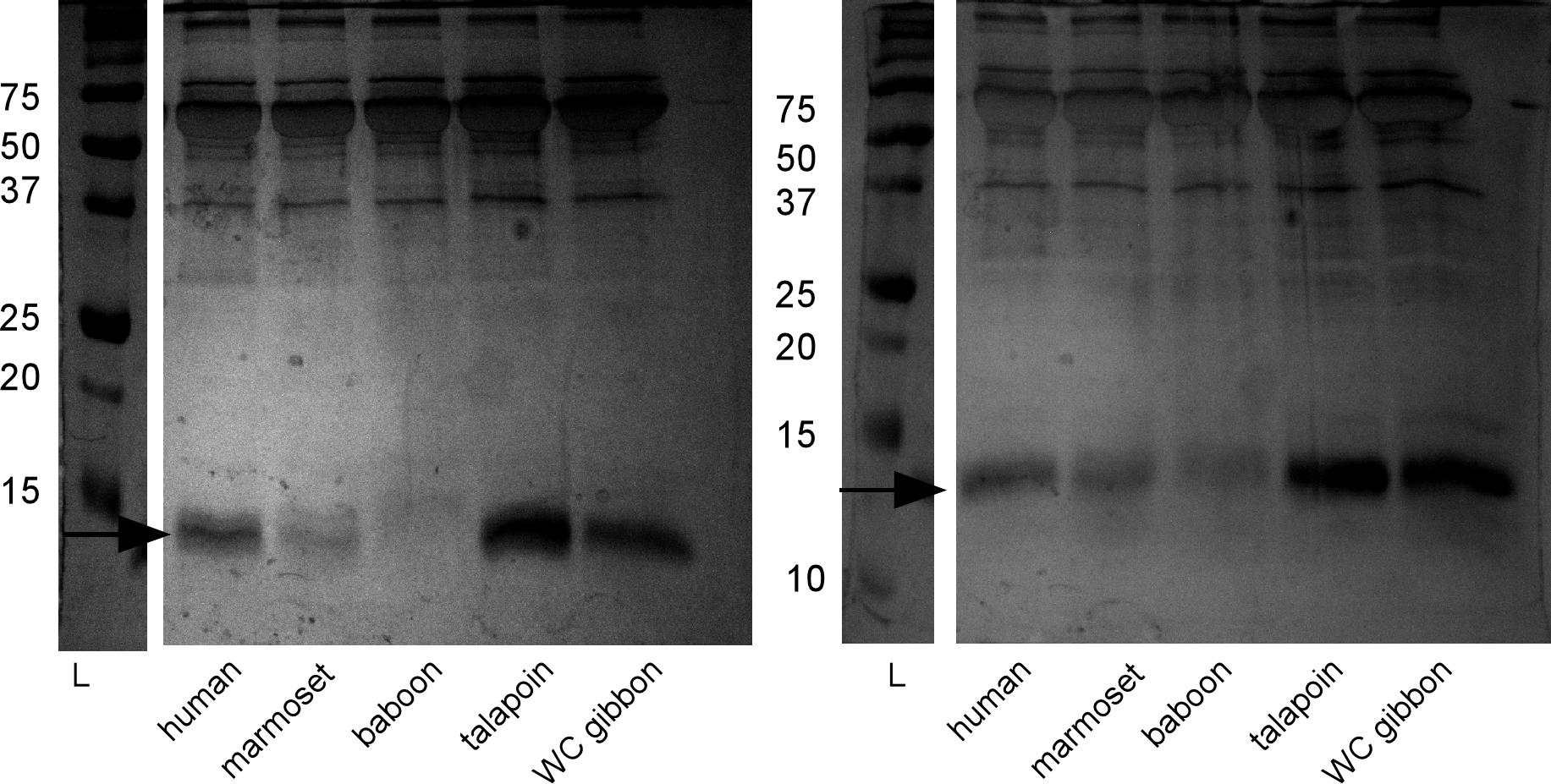

Supplement: FIG S2 [file mbo006184177sf2.tif]

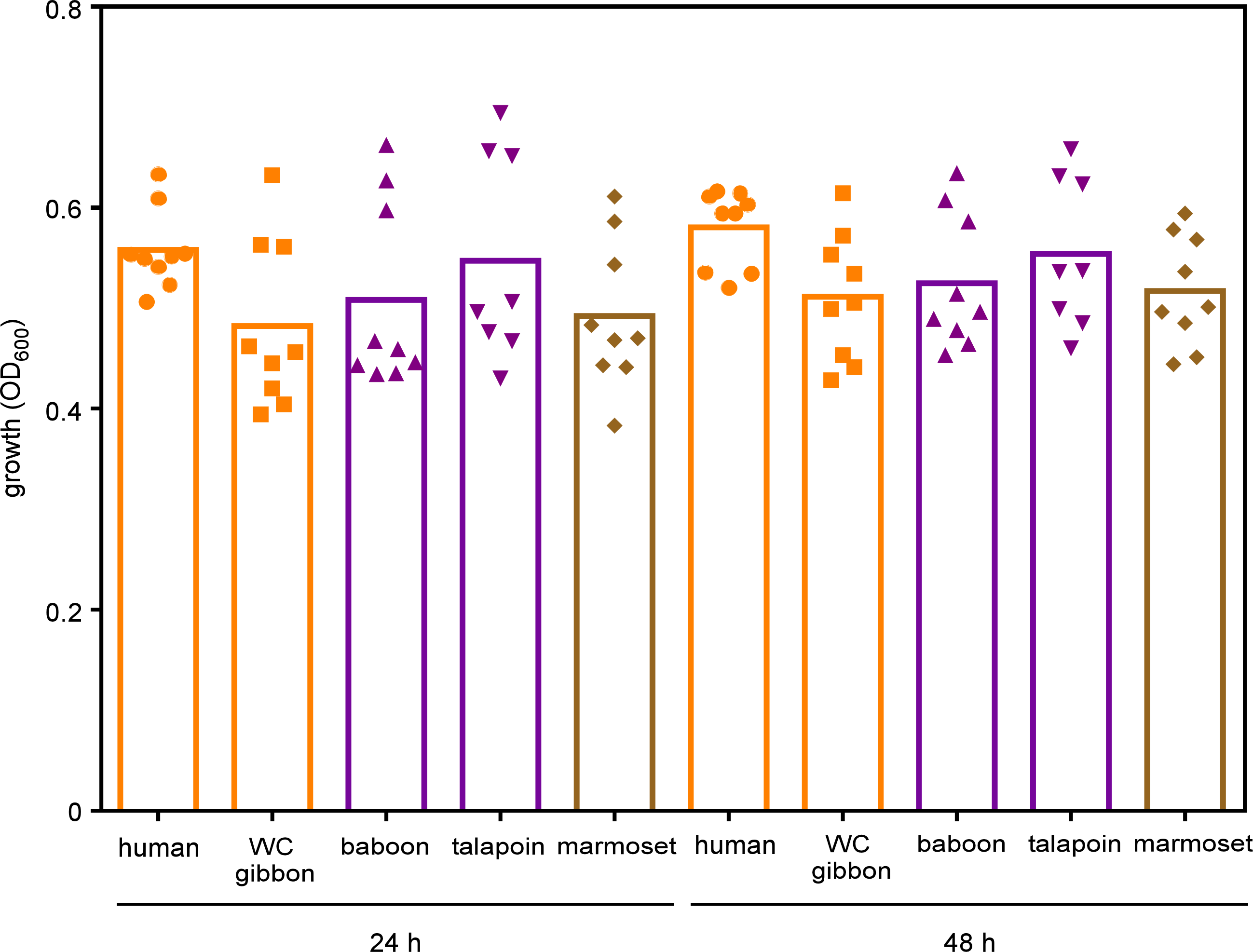

Supplement: FIG S3 [file mbo006184177sf3.tif]

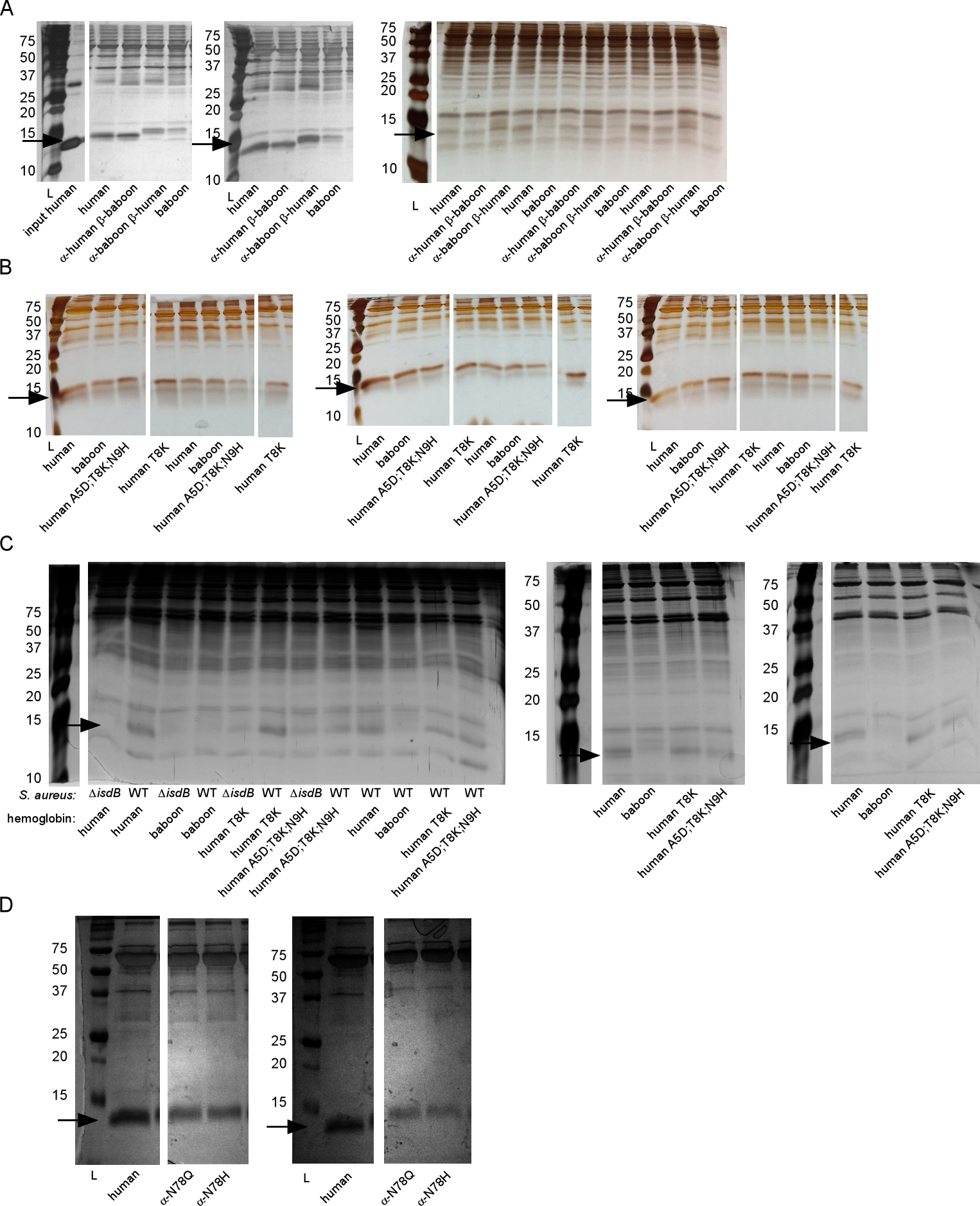

Supplement: FIG S4 [file mbo006184177sf4.tif]

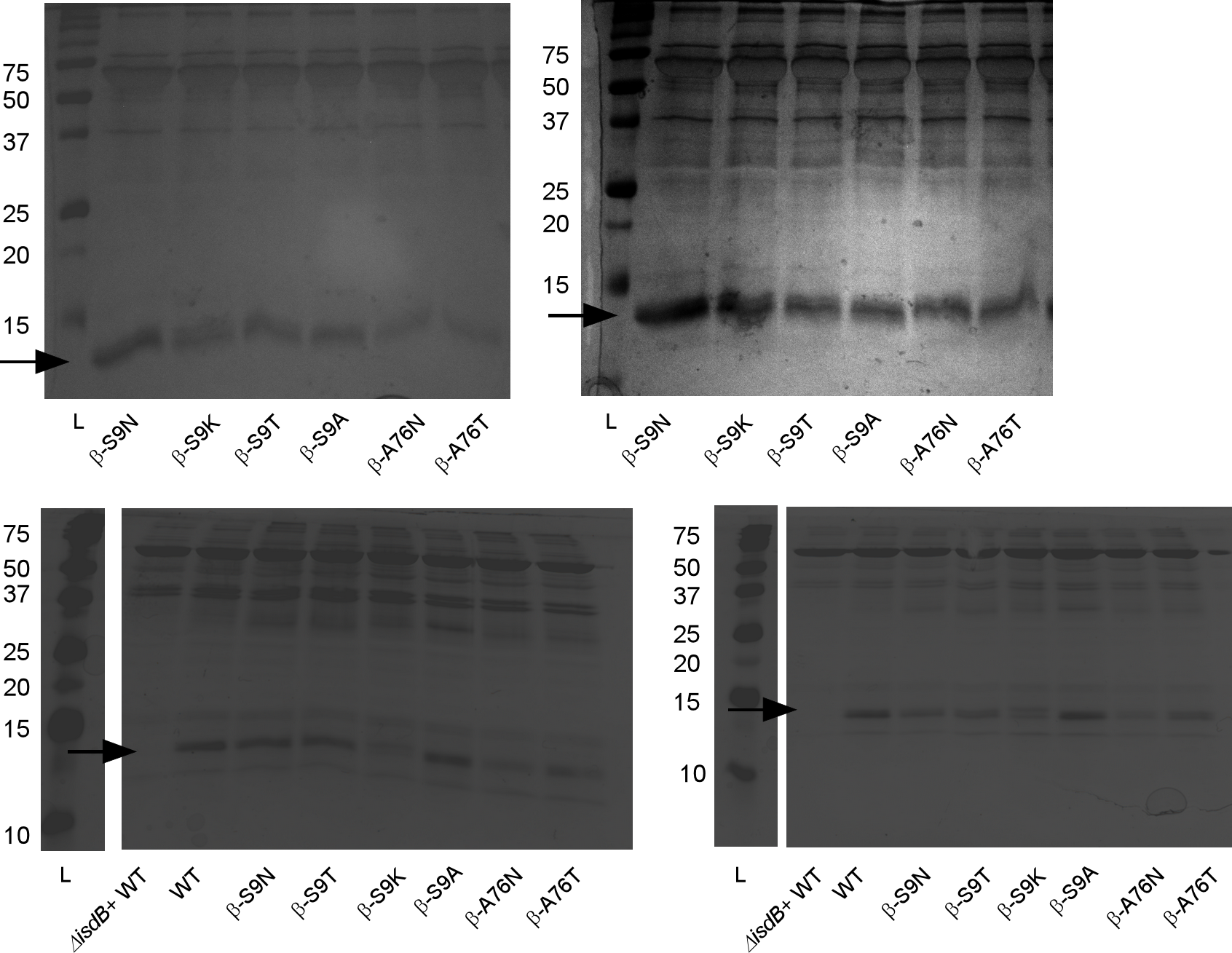

Supplement: FIG S5 [file mbo006184177sf5.tif]

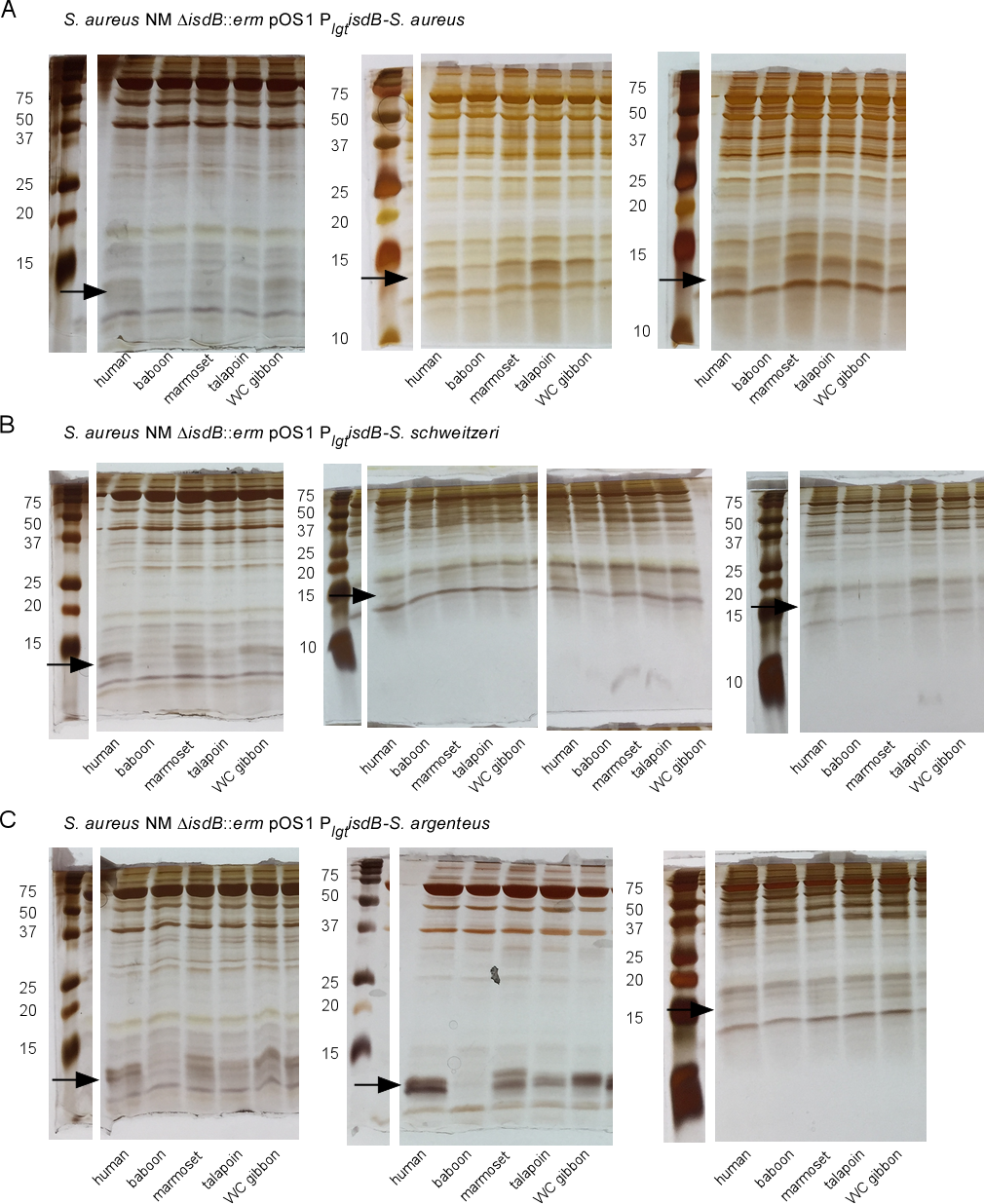

Supplement: FIG S6 [file mbo006184177sf6.tif]

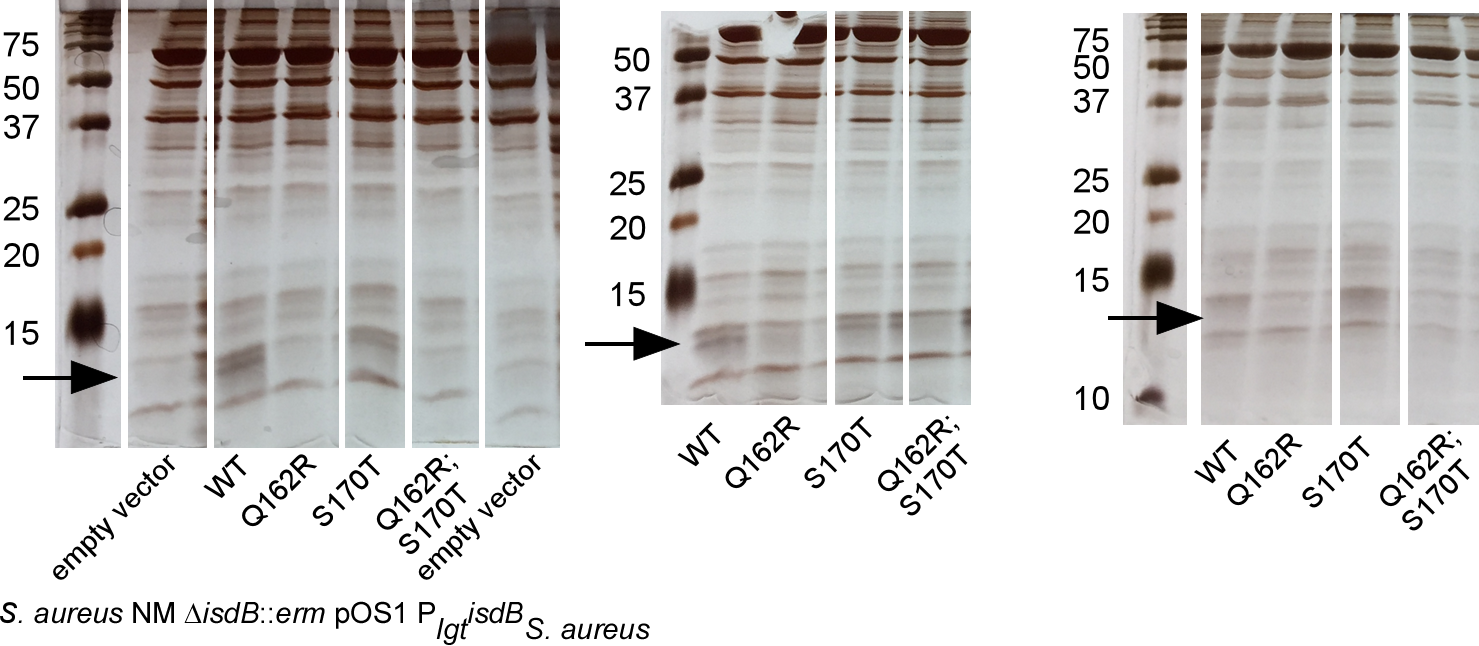

Supplement: FIG S7 [file mbo006184177sf7.tif]

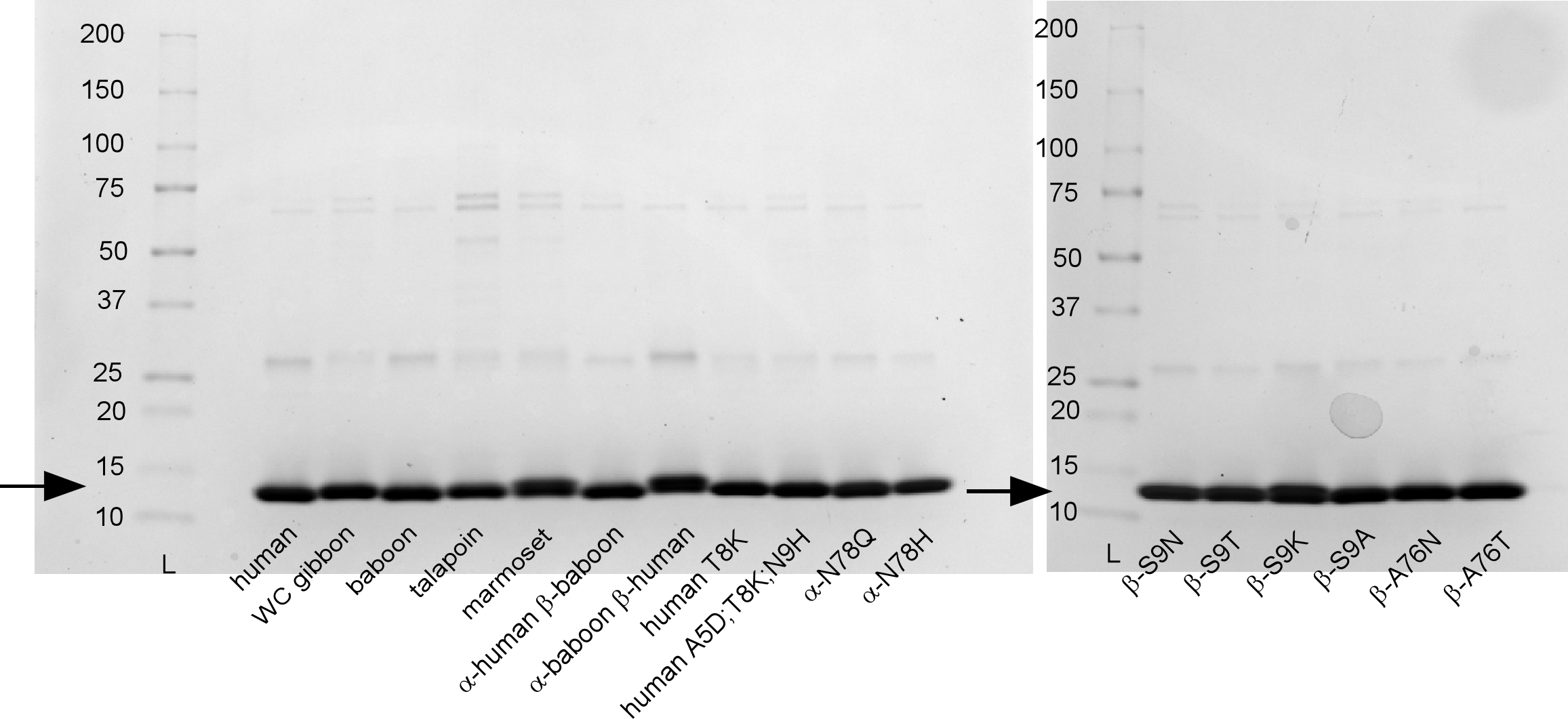

Supplement: FIG S8 [file mbo006184177sf8.tif]
