# Supplementary material for: Molecular Basis for the Evolution of Species-Specific Hemoglobin Capture by Staphylococcus aureus
Source: mBio. 2018 Nov 20;9(6):e01524-18. doi: 10.1128/mBio.01524-18 (PMC6247092; doi:10.1128/mBio.01524-18)
Supplement: TABLE S1 [file mbo006184177st1.docx]

| Table S1. Oligonucleotides used in this study. | | |
| --- | --- | --- |
| Primer name | Sequence | Description |
| AF327 | TTCACTAAGGAGGTTAATTAATGGTGCTGTCTCCTGCC | Forward primer for white-cheek gibbon and talapoin α-globin |
| AF328 | TCTAGATCATTAACGGTATTTGGAGGTCAGC | Reverse primer for white-cheeked gibbon and talapoin α-globin |
| AF329 | TACCGTTAATGATCTAGATAAGGAGGTAAATATATG | Forward primer for white-cheeked gibbon, baboon, talapoin, and marmoset β-globin |
| AF330 | GAGCCTTTCGTTTTATTTAAGCTTCATTAGTGGTACTTGTGG | Reverse primer for white-cheeked gibbon, baboon, and talapoin β-globin |
| AF331 | TTCACTAAGGAGGTTAATTAATGGTGCTGTCTCCTGAC | Forward primer for baboon α-globin |
| AF332 | TCTAGATCATTAACGGTATTTGGAGGTCAG | Reverse primer for baboon α-globin |
| AF333 | TTCACTAAGGAGGTTAATTAATGGTGCTGTCTCCCGCC | Forward primer for marmoset α-globin |
| AF334 | TCTAGATCATTAACGGTATTTGGAGGTCAGCAC | Reverse primer for marmoset α-globin |
| AF335 | GAGCCTTTCGTTTTATTTAAGCTTCATTAGTGGTACTTGTGAG | Reverse primer for marmoset β-globin |
| AF289 | CTGTCTCCGGCCGATAAAAAGAACGTTAAAGCTGCTTGG | Forward primer to create T8K in human α-globin |
| AF290 | CCAAGCAGCTTTAACGTTCTTTTTATCGGCCGGAGACAG | Reverse primer to create T8K in human α-globin |
| JC112 | TCTCCGGCCGATAAAAAGCACGTTAAAGCTGCTTGGGGT | Forward primer to create N9H in human α-globin using pHb0.0-human αT8K as template |
| JC113 | ACCCCAAGCAGCTTTAACGTGCTTTTTATCGGCCGGAGA | Reverse primer to create N9H in human α-globin pHb0.0-human αT8K as template |
| JC114 | TAATTAATGCTGTCTCCGGACGATAAAAAGCACGTTAAA | Forward primer to create A5D in human α-globin using pHb0.0-human αT8K;N9H as template |
| JC115 | TTTAACGTGCTTTTTATCGTCCGGAGACAGCATTAATTA | Reverse primer to create A5D in human α-globin using pHb0.0-human αT8K;N9H as template |
| AF291 | CACGTTGATGATATGCCGCAGGCGTTGTCTGCTCTGTC | Forward primer to create N78Q in human α-globin |
| AF292 | GACAGAGCAGACAACGCCTGCGGCATATCATCAACGTG | Reverse primer to create N78Q in human α-globin |
| AF293 | CACGTTGATGATATGCCGCACGCGTTGTCTGCTCTGTC | Forward primer to create N78H in human α-globin |
| AF294 | GACAGAGCAGACAACGCGTGCGGCATATCATCAACGTG | Reverse primer to create N78H in human α-globin |
| AF303 | CTGACTCCGGAAGAAAAAAACGCGGTTACTGCTCTG | Forward primer to create S9N in human β-globin |
| AF304 | CAGAGCAGTAACCGCGTTTTTTTCTTCCGGAGTCAG | Reverse primer to create S9N in human β-globin |
| AF307 | CTGACTCCGGAAGAAAAAACCGCGGTTACTGCTCTG | Forward primer to create S9T in human β-globin |
| AF308 | CAGAGCAGTAACCGCGGTTTTTTCTTCCGGAGTCAG | Reverse primer to create S9T in human β-globin |
| AF305 | CTGACTCCGGAAGAAAAAAAGGCGGTTACTGCTCTG | Forward primer to create S9K in human β-globin |
| AF306 | CAGAGCAGTAACCGCCTTTTTTTCTTCCGGAGTCAG | Reverse primer to create S9K in human β-globin |
| AF309 | CTGACTCCGGAAGAAAAAGCCGCGGTTACTGCTCTG | Forward primer to create S9A in human β-globin |
| AF310 | CAGAGCAGTAACCGCGGCTTTTTCTTCCGGAGTCAG | Reverse primer to create S9A in human β-globin |
| AF313 | GCTTTCTCTGACGGTCTGAATCACCTGGACAACCTG | Forward primer to create A76N in human β-globin |
| AF314 | CAGGTTGTCCAGGTGATTCAGACCGTCAGAGAAAGC | Reverse primer to create A76N in human β-globin |
| AF311 | GCTTTCTCTGACGGTCTGACTCACCTGGACAACCTG | Forward primer to create A76T in human β-globin |
| AF312 | CAGGTTGTCCAGGTGAGTCAGACCGTCAGAGAAAGC | Reverse primer to create A76T in human β-globin |
| pHb0.0_for | CGACTGGAAAGCGGGCAG | Forward sequencing primer for pHb0.0 |
| pHb0.0_rev | GCATTGTTAGATTTCATACACG | Reverse sequencing primer for pHb0.0 |
| JC343 | AAATACAATTGAGGTGAACATATGATGAACAAACAGCAAAAAG | Forward primer to clone *S. aureus isdB* into pOS1 P*_lgt_* by Hi-fi assembly |
| JC344 | AAACACTACCCCCTTGTTTGGATCCTTAGTTTTTACGTTTTCTAGGTAATAC | Reverse primer to clone *S. aureus isdB* into pOS1 P*_lgt_* by Hi-fi assembly |
| JC218 | AAATACAATTGAGGTGAACATATGATGAACAAACAGCAAAAAG | Forward primer to clone *S. schweitzeri isdB* into pOS1 P*_lgt_* by Hi-fi assembly |
| JC219 | AAACACTACCCCCTTGTTTGGATCCTTAGTTTTTACGTTTTCTAGGTAATAC | Reverse primer to clone *S. schweitzeri isdB* into pOS1 P*_lgt_* by Hi-fi assembly |
| JC216 | AAATACAATTGAGGTGAACATATGATGAACAAACAGCAAAAAG | Forward primer to clone *S. argenteus isdB* into pOS1 P*_lgt_* by Hi-fi assembly |
| JC217 | AAACACTACCCCCTTGTTTGGATCCTTAGTTTTTACGTTTTCGAGG | Reverse primer to clone *S. argenteus isdB* into pOS1 P*_lgt_* by Hi-fi assembly |
| JC317 | TTATGCAAGTACTGTTAAACCTG | Forward primer, creates S170T in pOS1 P*_lgt_isdB _aureus_* |
| JC318 | TGATAAAACTGTTGAGTTCC | Reverse primer, creates S170T in pOS1 P*_lgt_isdB _aureus_* |
| JC315 | AGATGGAACTAGACAGTTTTATCATTATG | Forward primer, creates Q162R in pOS1 P*_lgt_isdB _aureus_* |
| JC316 | TTCTTTTTCATTTCAAAATCAATTG | Reverse primer, creates Q162R in pOS1 P*_lgt_isdB _aureus_* |
| JC319 | TTATGCAAGTACTGTTAAACCTGCTAGAGTTATTTTC | Forward primer, simultaneously creates Q162R;S170T in pOS1 P*_lgt_isdB _aureus_* |
| JC320 | TGATAAAACTGTCTAGTTCCATCTTTCTTTTTCATTTC | Reverse primer, simultaneously creates Q162R; S170T in pOS1 P*_lgt_isdB _aureus_* |
| JC228 | TAAGAAGAGATGTAAGAGTAGGG | pOS1 P*_lgt_* forward sequencing primer |
| JC229 | GGGGGAAACACTACCCCCTTG | pOS1 P*_lgt_* reverse sequencing primer |
